# Effectiveness and safety of moderate-intensity aerobic water exercise during pregnancy for reducing use of epidural analgesia during labor: protocol for a randomized clinical trial

**DOI:** 10.1186/s12884-018-1715-3

**Published:** 2018-04-11

**Authors:** Araceli Navas, Catalina Artigues, Alfonso Leiva, Elena Portells, Aina Soler, Antonia Cladera, Silvia Ortas, Margarita Alomar, Marina Gual, Concepción Manzanares, Marina Brunet, Magdalena Julià, Lidia López, Lorena Granda, Miquel Bennasar-Veny, Mari Carmen Carrascosa

**Affiliations:** 1 Baleares Health Services-IbSalut, Hospital de Son Espases, 07005 Palma, Spain; 2 Pont D’Inca- Marratxí Health Care Center. Baleares Health Services-IbSalut, 07005 Palma, Spain; 3 Primary Care Research Unit of Mallorca. Baleares Health Services-IbSalut, 07005 Palma, Spain; 4 Instituto de Investigación Sanitaria de Palma(IdISPa), 07010 Palma, Spain; 5 Muntanya- Marratxí Health Care Center. Baleares Health Services-IbSalut, 07005 Palma, Spain; 6 Santa María Health Care Center. Baleares Health Services-IbSalut, 07005 Palma, Spain; 7 S’Excorxador Health Care Center. Baleares Health Services-IbSalut, 07005 Palma, Spain; 8 Can Pastilla Health Care Center. Baleares Health Services-IbSalut, 07005 Palma, Spain; 9 Soller Health Care Center. Baleares Health Services-IbSalut, 07005 Palma, Spain; 10 Rafal Nou Health Care Center. Baleares Health Services-IbSalut, 07005 Palma, Spain; 11 Santa Ponça Health Care Center. Baleares Health Services-IbSalut, 07005 Palma, Spain; 12 Alcudia Health Care Center. Baleares Health Services-IbSalut, 07005 Palma, Spain; 13 Baleares Health Services-IbSalut, Hospital Comarcal de Inca, 07005 Palma, Spain; 14 Baleares Health Services-IbSalut, Hospital de Manacor, 07005 Palma, Spain; 15 Department of Nursing and Physiotherapy, Research Group on Evidence, Lifestyles & Health, University of the Balearic Islands, 07122 Palma, Spain; 16 Son Ferriol Health Care Center. Baleares Health Services-IbSalut, 07005 Palma, Spain

**Keywords:** Exercise, Delivery, Postnatal depression, Epidural anesthesia, Caesarian, Natural childbirth

## Abstract

**Background:**

Epidural analgesia during labor can provide effective pain relief, but can also lead to adverse effects. The practice of moderate exercise during pregnancy is associated with an increased level of endorphins in the blood, and this could also provide pain relief during labor. Aerobic water exercises, rather than other forms of exercise, do not negatively impact articulations, reduce edema, blood pressure, and back pain, and increase diuresis. We propose a randomized controlled trial (RCT) to evaluate the effectiveness and safety of a moderate water exercise program during pregnancy on the need for epidural analgesia during labor.

**Methods:**

A multi-center, parallel, randomized, evaluator blinded, controlled trial in a primary care setting. We will randomised 320 pregnant women (14 to 20 weeks gestation) who have low risk of complications to a moderate water exercise program or usual care.

**Discussion:**

The findings of this research will contribute toward understanding of the effects of a physical exercise program on pain and the need for analgesia during labor.

**Trial registration:**

ISRCTN Registry identifier:14097513 register on 04 September 2017. Retrospectively registered.

## Background

Epidural analgesia is one of the most effective methods for reducing labor pain [1–3]. This technique is used for pain relief in labor in 25% of women in the United Kingdom [4], 58% of women in the United States, [5] and 58 to 61% of women in Spain [6, 7].

Although epidural analgesia provides effective pain relief, it may lead to complications or adverse effects, with maternal and fetal morbidities. The risk of accidental dural puncture due to epidural catheter insertion is 1.5%, and approximately half of these cases have postdural puncture headache [8–10]. There are many other complications related to epidural analgesia and these including: high block, which can lead to maternal hypotension, respiratory arrest, and loss of consciousness; epidural hematoma, a serious complication that can cause compression or ischemia of the spinal cord and subsequent paralysis; and local anesthetic toxicity due to an inadvertent intravascular injection that provokes respiratory and cardiac arrest [11].

In addition, some studies reported an association between epidural use and prolonged labor, increased use of instrumental deliveries, increased use of Caesarean sections, [11–13] and increased utilization of hospital resources [14]. Many caregivers are currently searching for non-pharmacological methods to control pain during labor, [15–18] and a major report from the World Health Organization has encouraged this approach [19]. Non-pharmacologic approaches to pain management include a wide variety of techniques that aim to reduce the physical sensations of pain and to prevent suffering by addressing the psychoemotional and spiritual components of care. Some studies suggest that exercise increases beta-endorphin levels, provides pain relief during labor, and reduces the need for epidural analgesia [20–23]. However the results of studies on reduced pain perception [24] due to increased levels of beta-endorphins at the time of delivery are inconclusive [24, 25].

Clinical practice guidelines (CPG) and national and regional strategies recommend exercise during pregnancy because it provides beneficial effects without adversely affecting the fetus. In particular, the American College of Obstetricians and Gynecologists (ACOG) guidelines from 2002 [26] and 2015 [27] and the Canadian Society of Gynecology and Obstetrics (SOGC) guidelines from 2003 [28] recommend exercise during pregnancy and the postpartum period. These guidelines suggest a minimum of 30 min of moderate daily exercise (about 150 min per week) for healthy pregnant women without obstetric complications. This exercise regimen can improve the health of active or sedentary women, with no risk to the fetus [29, 30].

Some observational studies reported that performance of exercise may help prevent and treat gestational diabetes and pre-eclampsia during pregnancy [31–33], and reduce the rates of Cesarean section, instrumental delivery, and postpartum recovery time. However, randomized clinical trials provided only limited support for these benefits [27].

Aerobic activity during pregnancy does not negatively impact preterm birth, newbornweight, but is related to a minor maternal weight gain [29, 34]. However, physical inactivity and excessive maternal weight are recognized risk factors for development of complications, such as gestational diabetes and maternal obesity [27]. A large cohort study of the relationship between exercise and pregnancy loss reported increased abortion in women performing aerobic high-impact exercise (jogging, ball games, and racquet sports) for more than 7 h per week, but no association between exercise and the risk of miscarriage after 18 weeks of gestation [35].

Aquatic-aerobic exercise has some advantages over other forms of exercise. These include reduced impact on articulations, less edema, increased diuresis, reduced arterial pressure, better control of body weight, less back pain, better temperature control, and lower probability of miscarriage [36]. Moreover, aquatic-aerobic exercise is not associated with risk of urinary or vaginal infections [27, 37–39]. Also, because aquatic-aerobic exercise is a group activity, compliance should be greater and there will be emotional interactions among women, and this may motivate the participants to develop healthier behaviors outside of exercise class [40, 41]. Moreover, aerobic water exercise could be an alternative to pain control during labor, reduce adverse effects such as dystocia, increase maternal satisfaction, and is likely to be readily accepted by pregnant women.

We propose a randomized clinical trial to analyze the effectiveness, safety, and cost of a moderate aerobic water exercise program on the use of epidural analgesia during labor.

### Objectives

The main objective of this study of women with uncomplicated pregnancies is to compare a standard antenatal care program with a program of aquatic-aerobic exercises of moderate intensity on the use of epidural analgesia during labor. The secondary objectives are to test the effect of the water exercise program on use of epidural analgesia before 6-cm cervical dilation, dystocic delivery, induction of labor, subjective satisfaction with the experience of childbirth, pain perception during the first stage of labor, duration of labor (first and second stages), and postpartum depression, and to assess the safety and cost-effectiveness of the aquatic-aerobic exercise program.

## Methods/design

**Fig. 1:**
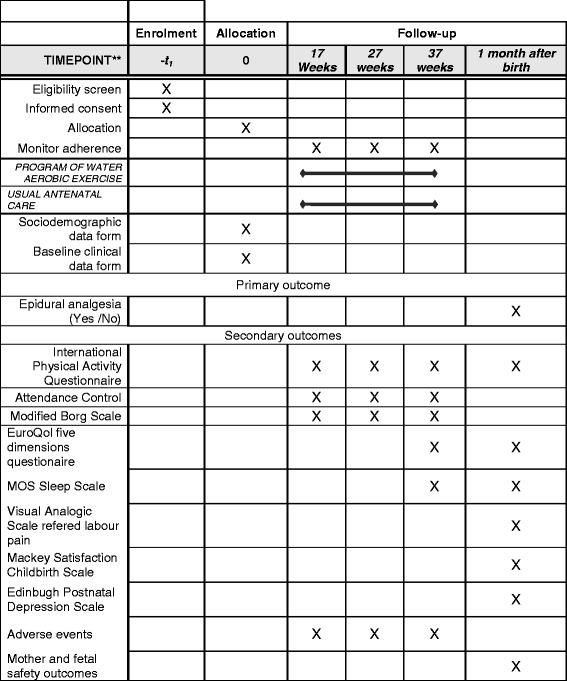
Study design and timeline

We designed a multi-center, parallel, randomized, evaluator-blinded, controlled clinical trial of 320 pregnant patients from primary care centers in Mallorca, Spain. Participants will be randomly allocated to a moderate aquatic-aerobic exercise group or a usual antenatal care group. Figure 1 summarizes the study design and timeline. Participants will be monitored from recruitment (at 14 to 20 weeks gestation) until the end of pregnancy, and then followed for 1 month after birth.

### Study population

Women will be recruited from 5 primary care centers in Mallorca, Spain. The participating primary care centers are all in a health district of a tertiary obstetric metropolitan hospital which has facilities for deliveries at 28 weeks or more. Women who are willing and able to comply with the requirements of the study will be invited to participate. Gestational age will be determined by the last menstrual period and/or by early ultrasound.

#### Inclusion criteria

- Pregnant women, who are 18 and 40 years-old and pregnant for 14 to 20 weeks.
- Pregnancy at low risk of complications (i.e. all women have singleton pregnancies, and none have medical, obstetric, or psychiatric problems).

#### Exclusion criteria

- Severe and poorly controlled hypertension, type 1 diabetes, or asthma.
- Hemodynamically significant heart disease, recent episode of deep venous thrombosis, hepatic insufficiency, or renal failure.
- Diagnosed mental illness or contraindications for physical activity.
- Multiparity (≥6 pregnancies), recurrent spontaneous miscarriages (≥3), incompetent cervix, increased risk of premature labor, persistent second or third trimester bleeding, uncontrolled gestational diabetes, severe isoimmunization, or planned Caesarean section.
- Severe anemia (hemoglobin < 9 mg/dL), recurrent urinary tract or vaginal infection, BMI above 35 or below 17, active and heavy smoker (> 20 cigarettes/day), any drug use or abuse, chronic infectious disease (HIV, hepatitis B, hepatitis C).
- Not being able to swim, communication difficulties, or unwillingness to provide informed consent.

### Sample size

The sample size calculation is based on the primary outcome measure and the primary analysis for the intention-to-treat population. Obstetric epidural analgesia in vaginal deliveries is estimated as 61% [42, 43]. Thus, we estimate a total reduction of at least 20% in the use of obstetric epidural analgesia in the intervention group based on a secondary analysis of a previous randomized clinical trial [21]. We adjusted the sample size for an estimated follow-up loss of 15% and a 0.05 two-sided level of significance (α = 5%).

Women in the control group might decide to attend other facilities to practice water exercise, swimming, or other physical activities. Thus, we increased the sample size by 15% to account for increased performance of exercise by women in the usual care group, so the final sample size will be 160 for each group (320 women total).

### Procedures, recruitment, randomization, and collection of baseline data

Each participating primary care center will have at least one midwife from the research team invite potential candidates to participate in the clinical trial and perform all procedures. Willing participants who meet the eligibility criteria will be enrolled after reading and signing an informed consent agreement. Then, entry details will be recorded on a trial entry form, and they will be randomized to the aquatic-aerobic exercise group or the usual antenatal care group. The 1:1 randomization will be performed by a researcher not involved with treatment allocation, and will be in balanced blocks of six.

### Intervention

The women randomized to the water aerobics group will participate in 45 min water aerobics classes held three times weekly in an indoor pool (28–30 °C) for 5 months. This procedure is based on recommendations of the American College of Sports Medicine [36], which proposes 3–5 classes per week, a training zone of 55–65% of maximum heart rate, classes of 20–60 min duration, maximum heart rate of 140 bpm, and maintenance of body temperature below 38 °C.

### Aquatic exercise

Four sets of exercise were developed for the study, each set of exercise include exercises that looks at the full work of muscle groups and include breathing and relaxation techniques. All exercises will be performed with coordinating breathing:

- Warm-up out of water (5 to 7 min).
- Warm-up in water (5 to 10 min).
- Moderate aquatic exercise (20 min).
- Breathing and relaxation exercises (5 min).
- Playful exercises (5 min).

The intervention will stop if any of the followings events appear during the trial: metrorrhagia, placenta previa, premature rupture of membranes, intrauterine growth retardation, severe anemia, or any contraindications to being physically active [27].

Women in the control group will receive standard antenatal care, and the customary information given by a midwife or general practitioner. They were not discouraged from exercising on their own.

### Follow up of women and infants

Midwifes will be responsible for data collection from the clinical history and from the questionnaires in all follow-up visits at gestation times of 17, 27, and 37 weeks. One month after birth, a follow-up visit will be used to assess maternal well-being, satisfaction with care, use of breast feeding, and early infant complications. Resource utilization and delivery of information regarding birth and infant outcomes will be obtained from the woman’s and infant’s case notes by the local research coordinator.

### Measurement of outcomes

#### Main outcome variables

The main outcome measure will be the incidence of epidural analgesia use during labor. This will be determined through review of the clinical history by a member of the research team who is blinded to the allocations.

#### Secondary outcome variables

##### Maternal medical outcomes

- Incidence of epidural analgesia use of epidural analgesia before 6-cm cervical dilation
- Method of delivery (normal spontaneous vaginal, assisted vaginal, or Caesarean section).
- Episiotomy or perineal tear.
- Morbidity: major complications (during the antepartum, intrapartum, and postpartum periods), poor labor progress, intrapartum maternal fever, type of membrane rupture.
- Pregnancy weight gain.
- Induction of labor.
- Robson group, cervical dilation, time of active labor, time of expulsive labor.

### Total labor pain

Total labor pain will be measured using a visual analogue scale (VAS). The VAS is one of the most used pain assessment instruments in research and clinical practice. Previous research indicted the VAS is reliable and sensitive for assessment of labor pain [44].

### Edinburgh postnatal depression scale (EPDS)

The EPDS is a questionnaire originally developed to assist in identifying possible symptoms of depression in women during the postnatal period. It also has adequate sensitivity and specificity for identification of depressive symptoms in the antenatal period, and is useful in identifying symptoms of anxiety. The Spanish language version of the EPDS has been validated [45].

This study will also collect data on health resource utilization and pregnancy outcome, and will record and compare the rates of pregnancy complications and perinatal outcomes in the two groups. The latter will be obtained (with informed consent) from the existing computerized obstetric database and include:

### EuroQol five dimension questionnaire (EQ-5D)

The EQ-5D will be used to assess quality of life. This is a standard instrument used to measure overall health status, and its has good validity and reliability for individuals with various health conditions [46]. The EQ-5D is a preference-based HRQL measure with one question for each of the five dimensions that include mobility, self-care, usual activities, pain/discomfort, and anxiety/depression. The EQ-5D questionnaire also includes a Visual Analog Scale (VAS), by which respondents can report their perceived health status with a grade ranging from 0 (the worst possible health status) to 100 (the best possible health status).

#### Neonatal medical outcomes

Signs of intrapartum fetal distress (fetal heart rate abnormalities, birth weight, gestational age, Apgar score, pH, umbilical cord blood).

#### Resource utilization outcomes

Oxytocin/prostaglandin induction, oxytocin augmentation, amniotomy, narcotic analgesia, other type of pain relief (tub/bath/shower), antibiotic use, continuous electronic fetal monitor, need for maternal blood transfer, maternal length of stay (48 h vs. more than 48 h), admission to a neonatal intensive care unit, need for positive pressure ventilation, sepsis work-up and treatment, and neonatal readmission (< 28 days-old), non-delivery admissions and emergency room: antepartum hospital admission, emergency room without admission, postpartum maternal readmission (30 days post-partum), use of comprehensive perinatal services program, and non-planned visits to a primary care center.

#### Resource utilization outcomes

##### Sleep interference

The Medical Outcomes Study (MOS) sleep scale will be used to assess the quality and quantity of sleep. This questionnaire has 12 items that assess the key constructs of sleep. It is a self-administered questionnaire in which patients are asked to recall sleep-related activities over the past four weeks. There is scoring in six domains: sleep disturbance (4 items), snoring (1 item), awakening with shortness of breath or a headache (1 item), quantity of sleep (1 item), optimal sleep (1 item), sleep adequacy (2 items), and daytime somnolence (3 items) [47].

### International physical activity questionnaire-short form (IPAQ-SF)

The IPAW-SF will be used to assess physical activity in multiple domains, including leisure time, domestic and gardening (yard) activities, and work-related and transportation-related activities. The items in the questionnaire are structured to provide separate scores for walking, moderate-intensity activity, and vigorous-intensity activity, as well as a combined total score that describes overall level of activity. Computation of the total score requires summation of the duration (min) and frequency (days) of walking, moderate-intensity activity, and vigorous-intensity activity. This questionnaire is available in multiple languages, and a validated Spanish version will be used [48].

### Modified Borg scale (MBS)

The MBS is a frequently used quantitative measure of perceived exertion during physical activity. A single question asks the patient to rate the impression of exercise intensity on a scale of 0 (not at all) to 10 (maximal) [49].

### Baseline data collection

To assess the comparability of the study groups, baseline demographic and medical information will be collected from all medical records at the time of enrollment. The clinical history of the mother will include date of birth, socio-economic status, parity, date of last menstrual period, estimated date of partum, risk factors during pregnancy, clinical antecedents, surgery and personal obstetric data, health-related habits, and preference for analgesia during delivery.

### Blinding

The nature of this study did not allow blinding of women and midwifes to group allocation. However, the outcome assessments will be conducted by external personnel (not involved in the study) who will be blinded to allocation. To avoid a potential Hawthorne effect, in which women in the intervention group change their behaviors (e.g. avoid use of epidural analgesia) because they know they are being observed, the women were not specifically informed that use of epidural analgesia was the main outcome measure, although they were completely informed of all the aims of the study. The statistician and data entry staff were also blinded to allocations.

### Statistical analysis

We will test for significant baseline differences in the control and intervention arms by use of descriptive analysis, with continuous variables summarized by means and standard deviations if they have normal distributions, and by medians and 25th and 75th percentiles if they have non-normal distributions.

All analyses of the effectiveness and cost-effectiveness will consider the intention-to-treat population (i.e. all randomized patients, regardless of participation in any intervention session). This approach reduces bias that may occur when participants not receiving an assigned intervention are excluded from the analysis. All tests will be two-sided, and an α-value of 0.05 will be considered statistically significant.

We will compare the proportions of women in each arm who use epidural analgesia during labor, in which the null hypothesis is no difference between the groups. We will use the Chi-squared test in bivariate analysis, and will adjust for potential confounders (if any) using a logistic regression model.

We will estimate the relative and absolute risk reduction and the number needed to treat (NNT). All estimates will include 95% confidence intervals. The NNT will be calculated as the reciprocal of the difference between the proportion of women who required epidural analgesia during labor in the intervention and control arms.

The secondary measures of effectiveness, such as reduction of instrumental delivery, reduction of Caesarean section, and post-partum depression, will be evaluated by a Chi-squared test. We will use the Mann-Whitney U test to determine if there is a statistically significant reduction of pain during labor using a VAS.

The health economic analysis will be performed by calculating the incremental cost-effectiveness ratio (ICER) at 4 weeks from delivery. We will systematically collect data on use of all resources, including inpatient care, consultations with healthcare providers, use of drugs, and laboratory tests. To measure these effects, the EQ-5D scores will be used, and quality-adjusted life years (QALYs) will be determined. The ICER will be calculated as the difference in the mean costs of the 2 groups (C_1_ – C_T_) divided by difference in the mean effects of the 2 groups (E_1_ – E_T_):

$$ ICER=\frac{\overline{C_1}-\overline{C_T}}{\overline{E_1}-\overline{E_T}} $$

A nonparametric bootstrap procedure will be used to perform the uncertainty analysis for the ICER. This procedure considers the skewness of cost data, and the covariance of costs and QALYs. To control for possible confounding and to account for clustering, an alternative procedure (Net-Benefit Regression) will also be used. Cost-effectiveness acceptability curves will be presented to show statistical uncertainty. We will determine the safety of all interventions on the “safety population” and use “per protocol analysis” to compare adverse events of patients using the Chi- squared test.

### Safety

All adverse events will be recorded on a case report form. The study investigators will investigate the potential causal relationship of the study intervention with adverse events. All serious adverse outcomes, in the infant or the mother, during the course of this study will be reported to the ethics committee.

## Discussion

The management of labor pain is one of the main goals of maternity care, and non-pharmacologic approaches to labor pain are consistent with midwifery management and the choices of many women. There is conflicting evidence regarding the positive effect of exercise during pregnancy on the course of labor and delivery. Our RCT will provide important additional information on the effect of exercise during pregnancy and its impact in epidural use, as well as the rates of instrumental delivery, episiotomy, and induction.

A primary strength of this study is its rigorous methodology and the large numbers of health-care professionals and patients. These will strengthen the validity of the findings.

If the results of our study are conclusive, they might lead to changes in the standard care given to women during pregnancy, by providing an alternative to epidural analgesia for pain control during labor. The control of labor pain by an antenatal exercise program and without epidural analgesia could also improve the experience of childbirth for the mother, and could have a long-term effect on future pregnancies of the mother. There may be also substantial economic savings, due to a reduction of instrumental deliveries and post-partum depression. Our findings could also contribute to the development of better guidelines for good clinical practice in the care of pregnant women.

### Ethical considerations

This study will follow the principles outlined in the Declaration of Helsinki (with Tokyo 2004 amendment). All participants will provide written informed consent, and will be told that participation is voluntary and can be withdrawn at any time without any negative consequences concerning current or future medical treatments. Ethics approval has been obtained by the Primary Care Research Commision and the Institucional Review Board of the Balearic Islands Health Service (CEI-IB Ref. No: 2358/14).

All participants will be identified by a study number only, and the master code sheet linking names with numbers will be held securely and separately from the study data. To ensure that all information is secure, data records will only be accessible to research staff and authorized personnel. As soon as all follow-ups are completed, the data records will be de-identified. De-identified data will be used for the statistical analysis. All resulting publications will only include aggregate data.
